# Newly diagnosed diabetes mellitus is a risk factor for cardiocerebrovascular events in primary aldosteronism

**DOI:** 10.1007/s12020-022-03095-8

**Published:** 2022-07-29

**Authors:** Cui Zhang, Yiran Jiang, Tingwei Su, Lei Jiang, Weiwei Zhou, Xu Zhong, Luming Wu, Weiqing Wang

**Affiliations:** Department of Endocrine and Metabolic Diseases, Shanghai Institute of Endocrine and Metabolic Diseases, Ruijin Hospital, Shanghai Jiao Tong University School of Medicine, Shanghai, China

**Keywords:** Aldosterone, Newly diagnosed DM, Cardiovascular disease risk

## Abstract

**Objective:**

To explore the prevalence and clinical significance of newly diagnosed diabetes mellitus (DM) in patients with primary aldosteronism (PA). Investigating the risk factors for cardiocerebrovascular disease (CCVD) will guide strategies for reducing CCVD in patients with PA.

**Methods:**

We retrospectively included 729 PA patients without DM and conducted oral glucose tolerance tests.

**Results:**

We found that 15.0% of PA patients had newly diagnosed DM. The DM prevalence increased with elevated aldosterone levels [OR = 3.20 (1.77, 5.78), *P* value < 0.001]. The rate of CCVD in newly diagnosed diabetic PA patients was higher than that in nondiabetic PA patients at diagnosis (11.9% vs. 5.0%, *P* = 0.005). Furthermore, multivariate logistic analysis revealed that HT duration [1.055 (1.002,1.111), *P* = 0.041] and newly diagnosed DM [2.600 (1.072,6.303), *P* = 0.034] were significantly associated with CCVD in PA patients.

**Conclusion:**

The prevalence of newly diagnosed DM in PA patients was higher than that in the general population. Aldosterone level was an independent risk factor for DM not for CCVD. CCVD was correlated with longer HT duration and newly diagnosed DM. Therefore, it is crucial to screen DM at the diagnosis in PA patients.

## Introduction

Primary aldosteronism (PA) is a common secondary hypertension that accounts for 7.1% of resistant hypertension (HT) in China [1]. Excessive aldosterone is an independent risk factor for left ventricular hypertrophy, microalbuminuria and cardiocerebrovascular (CCV) events [2]. The prevalence of diabetes in China is 11.6% [3]. It is well known that diabetes alone could increase the risk of cardiovascular disease (CVD) [4]. PA comorbid with diabetes results in a higher risk of CVD than aldosteronism alone. It has been reported that diabetes itself increases the risks of CCV events and renal complications in patients with PA [5]. Therefore, screening for diabetes in PA patients is important, but few studies have analyzed new-onset diabetes in PA patients. The aim of this study was to determine the prevalence of newly diagnosed DM in PA patients and whether new-onset DM increases the CCV risk compared to PA in patients without DM.

Some studies have indicated that excess plasma aldosterone has a direct influence on glucose metabolism and lipid metabolism, resulting in impaired glucose metabolism and dyslipidemia [6–8]. However, some other studies reported inconsistent or even opposite results [9, 10]. Several mechanisms for the effect of plasma aldosterone on glucose and lipid metabolism in PA patients, including hypokalemia, reduced β-cell function, insulin receptor signaling in adipose tissue and hepatocytes and altered renal function, have been discussed [11–13]. The German Conn Registry and Augsburg (KORA)-F4 study found that PA patients with cortisol co-secretion had a higher prevalence of type 2 DM (T2DM) than PA patients without cortisol co-secretion. This indicates that cortisol co-secretion impairs glucose tolerance in peripheral tissues and impairs insulin-dependent glucose uptake in peripheral tissue [14]. However, this result needs to be validated in larger cohorts and other ethnicities. To address this and clarify the controversial results, we aimed to determine the prevalence of newly diagnosed DM in PA patients and explore the relationship between aldosterone and glucose metabolism in PA.

The purpose of this retrospective study was to explore the prevalence of newly diagnosed DM in PA. We also compared the clinical features of CCVD-positive and CCVD-negative PA patients and investigated the risk factors for CCVD.

## Patients and methods

### Study population

This retrospective study enrolled patients diagnosed with PA at the Shanghai Clinical Center for Endocrine and Metabolic Diseases in Ruijin Hospital Affiliated to Shanghai Jiaotong University School of Medicine from January 2008 to July 2018. PA was diagnosed according to the 2008 and 2016 PA guideline [15]. Patients with an aldosterone to renin ratio (ARR) > 30 (ng/dL)/(ng/ml·h) underwent a confirmatory test (iv saline load test). Post-saline infusion test (SIT) plasma aldosterone concentrations (PACs) > 10 ng/dL confirmed the diagnosis of PA [16–18]. Adrenal venous sampling (AVS) was performed by one experienced radiologist. Cannulation was considered successful if the cortisol adrenal vein/cortisol peripheral vein ratio was greater than 3 without adrenocorticotropic hormone (ACTH) stimulation. Lateralization was defined as the ratio of cortisol-corrected PAC of the dominant side/cortisol-corrected PAC of the nondominant side ratio of >2 without ACTH stimulation. A total of 1149 patients were diagnosed with PA. 101 of them were excluded due to a history of diabetes or the use of antidiabetic medications; 69 patients were excluded for incomplete data regarding metabolic parameters, 168 patients were excluded for not conducting AVS, and 82 patients were excluded for AVS failure. Finally, 729 participants, including 518 with unilateral PA and 211 with bilateral PA, were included. All patients underwent an oral glucose tolerance test for DM diagnosis. Newly diagnosed T2DM, impaired glucose tolerance (IGT), impaired fasting glucose (IFG), and normal glucose tolerance were defined according to the 2010 American Diabetes Association diagnostic criteria [19]. DM was diagnosed based on FPG ≥ 126 mg/dL (7.0 mmol/L) or 2-h glucose ≥200 mg/dL (11.1 mmol/L) or HbA1c ≥ 6.5%. Pre-DM including IFG and IGT, was diagnosed based on FPG 100 mg/dl (5.6 mmol/L) to 125 mg/dl (6.9 mmol/L) or 2-h PG 140 mg/dl (7.8 mmol/L) to 199 mg/dl (11 mmol/L) or A1C 5.7–6.4% [19]. The area under the curve (AUC) for plasma glucose was calculated by the trapezoidal method. CCVD include stroke (cerebral infarction or cerebral hemorrhage), coronary artery disease (myocardial infarction or angina pectoris), heart failure, atrial fibrillation.

### Data collected

All blood tests were performed after overnight fasting for at least 10 h in the hospital. Plasma samples for glucose and insulin detection were obtained at 0, 30, 60, 120 and 180 min after glucose loading, consistence with the 75-gram oral glucose tolerance test (OGTT). Plasma glucose concentrations and serum concentrations of triglycerides, total cholesterol, high-density lipoprotein cholesterol and low-density lipoprotein cholesterol were measured using an autoanalyzer (Beckman CX-7 Biochemical Autoanalyzer, Beckman Coulter, Brea, CA). Serum insulin was measured using an electrochemiluminescence assay (Roche Diagnostics, Basel, Switzerland). The level of hemoglobin A1c (HbA1c) was determined using the high-performance liquid chromatography method (VARIANT™ II and D-10™ Systems, BIO-RAD, Hercules, CA, USA). Serum aldosterone and plasma renin activity were measured by radioimmunoassay (RIA) following the manufacturer’s instructions (A Beckman Coulter Corp). Serum cortisol and serum ACTH were measured by immunoluminescence and RIA following the manufacturer’s instructions (A Beckman Coulter Corp.). All tests were performed in the College of American Pathologists (CAP)-accredited laboratory (No. 7217913).

### Statistical analysis

Continuous variables are expressed as medians (interquartile ranges 25–75); categorical variables are expressed as frequencies and percentages. The study population was divided into tertiles on the basis of serum aldosterone levels to explore any trends in metabolic variables, and linear regression was performed among the tertiles. The chi-square test was adopted to compare categorical variables. Logistic regression was used to evaluate the association between aldosterone and diabetes with three different models. The Mann-Whitney U test was used to compare the parameters of diabetic PA and nondiabetic PA. Multivariate logistic regression analyses were used to identify predictors associated with CCVD. A *P* value < 0.05 was considered to be statistically significant. SPSS (22.0) was used for all of the other analyses. The graphs were constructed with GraphPad (8.0).

## Results

### Cohort characteristics

A total of 729 patients diagnosed with PA were finally included in this study. The laboratory characteristics are listed in Table 1. In total, 109 patients were newly diagnosed with DM, accounting for 15%. 230 patients were diagnosed with prediabetes, accounting for 31.5%. In total, 46.5% of PA patients had glucose metabolism disorders. Forty-four patients reported a history of cardiocerebrovascular (CCV) events: 27 patients had stroke, 13 patients had coronary artery disease, 2 patients had heart failure and 2 patients had atrial fibrillation. According to AVS, 211 patients were diagnosed with bilateral hyperaldosteronism (BHA), and 518 patients were diagnosed with unilateral hyperaldosteronism (UHA). The two groups of patients were similar in age, sex, duration of HT, family history of HT, and blood pressure (*p* > 0.05). BHA patients had a higher BMI than UHA patients (*p* < 0.001). While cortisol and ACTH were similar between two groups (*p* > 0.05). FBG and PBG were slightly elevated in BHA without significance (*p* > 0.05). Insulin at 0 and 120 min were significantly higher in BHA than in UHA (*p* < 0.05). The prevalence of DM and abnormal glucose metabolism did not differ between the two groups.

**Table 1:** Clinical characteristics of cohort patients

	Cohort (*N* = 729)	BHA (*N* = 211)	UHA (*N* = 518)	*P* value*
Age (years)	47 (38, 55)	47 (38, 55)	48 (38, 54)	0.584
Gender (Male/Female)	372/357	111/110	261/257	0.587
Duration of HT (years)	6 (2,10)	5 (2,10)	6 (2,10)	0.608
Family history of HT (N,%)	430 (59.0%)	113 (53.6%)	317 (61.2%)	0.057
SBP (mmHg)	170 (159,180)	170 (152,180)	170 (160,180)	0.903
DBP (mmHg)	101 (100,115)	100 (100,120)	103 (100,110)	0.970
BMI (kg/m²)	24.24 (21.97,26.78)	25.04 (22.58,27.55)	23.96 (21.64,26.52)	0.001
Serum cortisol (ug/dL)	11.43 (8.77,14.44)	12.00 (9.05,15.14)	11.26 (8.71,14.01)	0.075
ACTH (pg/mL)	28.23 (20.50,39.75)	29.80 (20.95,43.10)	27.88 (20.38,39.03)	0.203
Serum K^+^ (mmol/L)	3.02 (2.80,3.33)	3.07 (2.82,3.36)	2.99 (2.76,3.29)	0.050
Serum Na^+^ (mmol/L)	142 (140,144)	142 (140,143)	142 (140,144)	0.028
PRA (ng/ml/h)	0.24 (0.08,0.58)	0.29 (0.10,0.64)	0.22 (0.08,0.53)	0.099
Aldosterone (ng/dL)	40.03 (26.21,60.65)	36.87 (24.92,50.40)	42.54 (27.11,63.83)	0.008
ARR (ng dl/ng ml h)	176.59 (64.54,540.01)	125.75 (51.56,493.02)	198.91 (72.99,581.36)	0.034
DM,N,%	109 (15.0%)	35 (16.6%)	74 (14.3%)	0.429
Abnormal glucose metabolism,N,%	339 (46.5%)	104 (49.3%)	235 (45.5%)	0.336
FBG (mmol/L)	5.10 (4.80,5.50)	5.19 (4.90,5.58)	5.10 (4.80,5.47)	0.059
PBG (mmol/L)	7.20 (5.90.9.20)	7.50 (6.02,9.83)	7.11 (5.90,8.95)	0.123
Insulin 0 min (μIU/ml)	6.06 (4.08,9.00)	7.15 (4.80,10.70)	5.78 (3.95,8.16)	<0.001
Insulin 120 min (μIU/ml)	47.06 (29.55,79.13)	54.37 (30.0,94.26)	44.48 (29.50,70.08)	0.004
HOMA-IR	1.39 (0.90,2.10)	1.64 (1.06,2.55)	1.29 (0.87,1.88)	<0.001
HbA1c	5.3 (5.0,5.6)	5.4 (5.2,5.8)	5.2 (4.9,5.5)	<0.001
History of CCV events (N,%)	44 (6.0%)	16 (7.6%)	28 (5.4%)	0.263

*P value compared between BHA and UHA

*BHA* bilateral hyperaldosteronism, *UHA* unilateral hyperaldosteronism, *HT* hypertension, *SBP* systolic blood pressure, *DBP* diastolic blood pressure, *BMI* body mass index, *ACTH* adreno-cortico-tropic-hormone, *K* potassium, *Na* sodium, *PRA* plasma renin activity, *ARR* aldosterone to renin ratio, *DM* diabetes mellitus, *CCV* cardiocerebrovascular

### The risk of diabetes and abnormal glucose metabolism increased with aldosterone

**Fig. 1:**
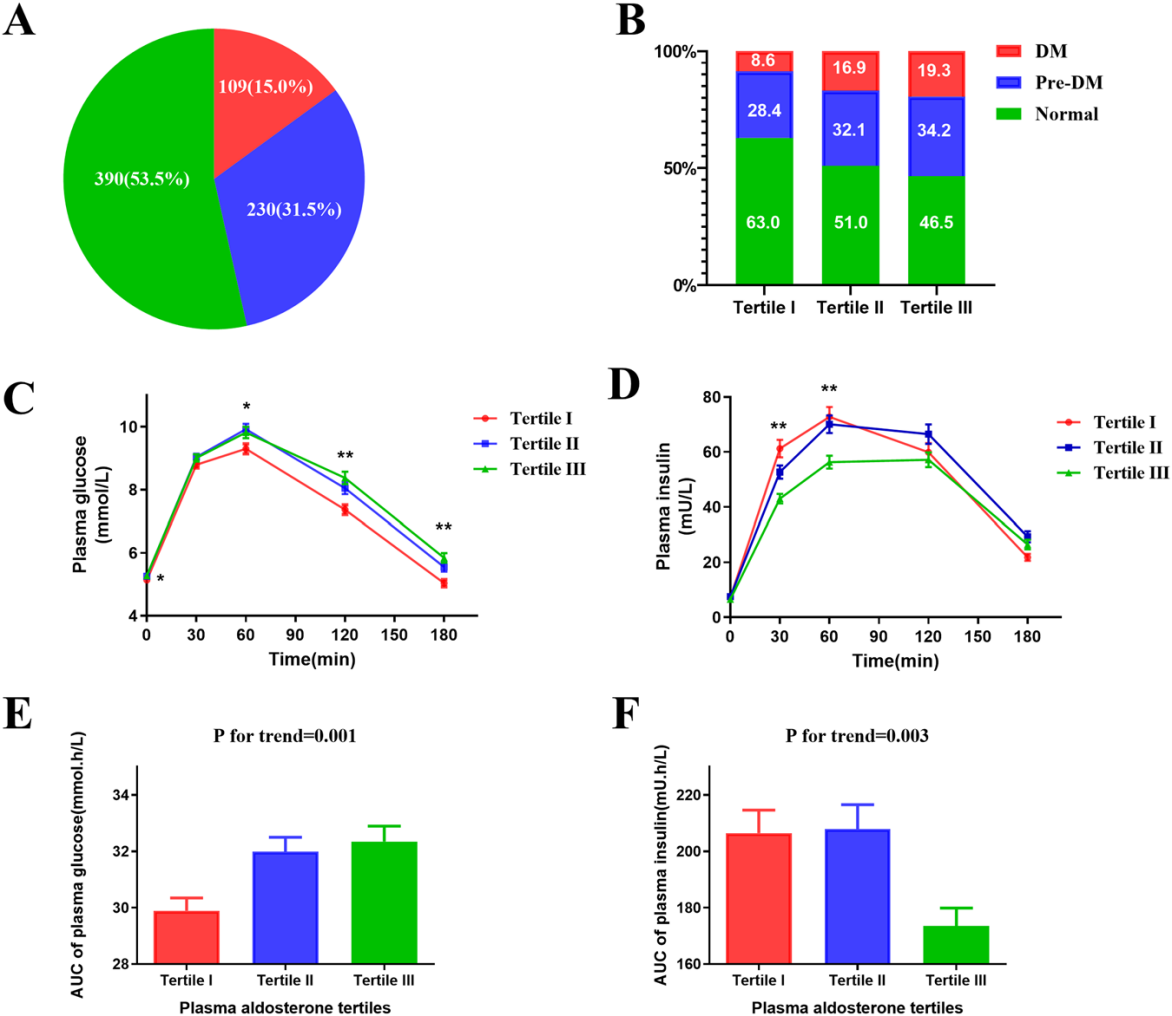
The prevalence of DM and pre-DM in the cohort patients and the glucose and insulin in the patients in three tertiles according to serum aldosterone level. **A** 15.0% patients of the total was DM and 31.4% patients of the total was pre-DM; **B** According to aldosterone level, patients were divided into three tertiles. In Tertile I, the prevalences of DM and pre-DM were 8.6% and 28.4%, repectively. In Tertile II, the prevalences of DM and pre-DM were 16.9% and 32.1%, respectively. In Tertile III, the prevalences of DM and pre-DM were 19.3% and 33.7%, respectively; (**C**) the plasma glucose in three tertiles at different time points (0 min, 30 min, 60 min, 120 min, 180 min) p for trend at 120 min and 180 min <0.001 and *p* for trend at 0 min and 60 min <0.05; (**D**). The serum insulin in three tertiles at different time points (0 min, 30 min, 60 min, 120 min, 180 min) *p* for trend at 30 min and 60 min <0.001; (**E**). The AUC of plasma glucose in three tertiles, p for trend = 0.001; (**F**) the AUC of serum insulin in three tertiles, p trend = 0.003

We divided the 729 PA patients into three groups according to serum aldosterone level. The aldosterone level of Tertile I was below 30.37 ng/dl, that of Tertile II ranged from 30.37 ng/dl to 51.12 ng/dl, and that of Tertile III was above 51.12 ng/dl. The three groups of patients were age-, sex- and blood pressure-matched (Supplementary Table S1). With elevated aldosterone levels, BMI 24.8 ± 3.7 vs. 24.6 ± 3.5 vs. 24.0 ± 3.3, P for trend=0.012 and serum K^+^ (3.15 ± 0.44 vs. 3.10 ± 0.42 vs. 2.86 ± 0.46, P for trend<0.001) decreased. Glucose metabolism was also influenced by aldosterone level. Both fasting blood glucose (FBG) and postprandial blood glucose (PBG) elevated with increasing aldosterone levels (5.13 ± 0.58 vs. 5.26 ± 0.70 vs. 5.27 ± 0.72, P for trend = 0.023; 7.36 ± 2.38 vs. 8.05 ± 2.92 vs. 8.37 ± 3.11, P for trend < 0.001, respectively). We also analyzed the AUCs for glucose and insulin according to the OGTT test in the three tertiles and found that with increasing aldosterone levels, the AUC for glucose increased and the AUC for insulin level decreased significantly (Fig. 1). We then compared the glucose and insulin levels at different time points (0 min, 30 min, 60 min, 120 min and 180 min) and found that the glucose level elevated with increasing aldosterone and that insulin decreased with increasing aldosterone.

The diagnosis rates of DM were 8.6%, 16.9%, and 19.3%, respectively, which significantly elevated with increasing serum aldosterone levels (P for trend = 0.001). The diagnosis rates of abnormal glucose metabolism (DM and pre-DM) were 37.0%, 49.0%, and 53.5%, respectively (*P* for trend < 0.001), which means that newly diagnosed DM and abnormal glucose metabolism rate increased significantly with increasing aldosterone tertiles. As shown in Table 2, we evaluated the correlation between aldosterone tertiles and the prevalence of DM with a logistic regression model. We observed significantly higher risks of diabetes with higher levels of aldosterone in Tertile II and Tertile III (OR = 2.15, 95% CI = 1.23–3.75; OR = 2.54, 95% CI = 1.46–4.39, respectively). After adjustments in Model 1 (age, sex and BMI) and Model 2 [age, sex, BMI, systolic blood pressure (SBP), diastolic blood pressure (DBP), duration of HT and serum cortisol], there were still significantly elevated risks of diabetes with increased levels of aldosterone (P for trend < 0.001). Abnormal glucose metabolism was significantly elevated with increasing aldosterone tertiles (OR = 1.63, 95% CI = 1.14–2.34; OR = 1.96, 95% CI 1.36–2.81). After adjustment in Model 1 (age, sex and BMI) and Model 2 (age, sex, BMI, SBP, DBP, duration of HT and serum cortisol), the prevalence of diabetes and abnormal glucose metabolism elevated significantly with increasing aldosterone (P for trend < 0.001).

**Table 2:** Association analysis of DM and tertiles of aldosterone

	Tertile I	Tertile II	Tertile III	P for trend
DM				
Number	21	41	47	
Unadjusted	1	2.15 (1.23,3.75)	2.54 (1.46,4.39)	0.001
Model 1*	1	2.62 (1.45,4.73)	3.24 (1.81,5.80)	<0.001
Model 2*	1	2.40 (1.32,4.36)	3.20 (1.77,5.78)	<0.001
Abnormal glucose metabolism				
Number	90	119	130	
Unadjusted	1	1.63 (1.14,2.34)	1.96 (1.36,2.81)	<0.001
Model 1*	1	1.94 (1.32,2.86)	2.40 (1.63,3.55)	<0.001
Model 2*	1	1.89 (1.27,2.80)	2.31 (1.55,3.43)	<0.001

^*^Model 1: adjusted for age sex BMI; Model 2: adjusted for age, sex, BMI, SBP, DBP, Duration of HT and Serum cortisol

*DM* diabetes mellitus, *SBP* systolic blood pressure, *DBP* diastolic blood pressure, *HT* hypertension

### Cardiocerebrovascular events increased in PA patients with newly diagnosed DM

**Fig. 2:**
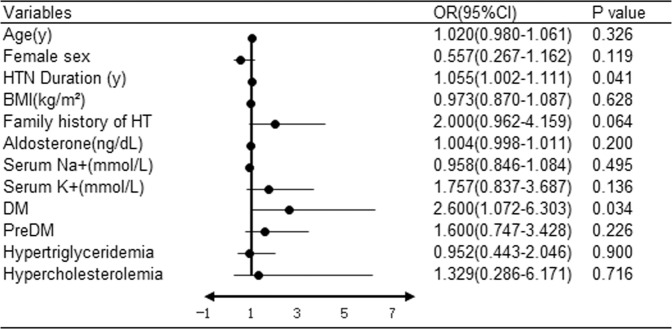
Effects of selected variables on differences between CCVD–positive PA patients and CCVD-negative PA patients

We compared parameters between PA patients with newly diagnosed DM and without DM (Table 3). We found that PA patients with newly diagnosed DM were older, male predominated, and had a longer duration of HT and higher BMI than PA patients without DM (*P* < 0.001), while blood pressure values were similar between the two groups. The aldosterone level was significantly higher in PA patients with DM; accordingly, serum sodium was higher and serum potassium was lower in these patients. However, plasma renin activity was significantly higher in patients with newly diagnosed DM than those without DM. We also compared the prevalence of CCV events in PA patients with newly diagnosed DM and without DM. The prevalence of CCVD was significantly higher in PA patients with DM than in those without DM (11.9% vs. 5.0%, *P* = 0.005). The prevalence of heart failure among PA patients with DM was much higher than that among PA patients without DM. We compared CCVD-positive and CCVD-negative PA patients (Supplementary Table S2) and found that CCVD-positive patients tended to be older (*P* = 0.001) and male predominated (*P* < 0.05), had a longer hypertension duration (*P* < 0.001), a more family history of HT (*P* < 0.05) and a higher DM prevalence (*P* = 0.005) than CCVD-negative patients. Furthermore, logistic regression analysis was performed to determine the factors affecting the presence of CCVD in PA patients (Fig. 2). We found HT duration [1.055 (1.002,1.111), *p* = 0.041] and newly diagnosed DM [2.600 (1.072,6.303), *p* = 0.034] were factors significantly associated with CCVD.

**Table 3:** The CCVD evaluation of DM and non-DM PA patients

	PA with DM (*N* = 109)	PA without DM (*N* = 620)	*P* value
Age(years)	52 (44,58)	46 (37,54)	<0.001
Gender(Male/Female)	79/30	293/327	<0.001
Duration of HT (years)	10 (4,14)	5 (2,10)	<0.001
BAH/UHA	35/74	176/444	0.429
Family history of HT	69 (63.3%)	361 (58.2%)	0.320
SBP(mmHg)	170 (155,190)	170 (160,180)	0.513
DBP(mmHg)	108 (97,120)	100 (100,115)	0.546
BMI(kg/m²)	26.23 (24.44,28.25)	23.86 (21.62,26.44)	<0.001
Serum K^+^(mmol/L)	2.91 (2.65,3.19)	3.04 (2.81,3.34)	0.001
Serum Na^+^ (mmol/L)	143 (141,144)	142 (140,144)	0.011
PRA(ng/ml/h)	0.37 (0.16,0.84)	0.22 (0.08,0.51)	<0.001
Aldosterone(ng/dL)	45.58 (32.64,66.36)	39.28 (25.07,59.19)	0.002
CCVD(%)	13 (11.9%)	31 (5.0%)	0.005
Coronary artery disease	4 (3.7%)	9 (1.5%)	0.107
Herat failure	2 (1.8%)	0 (0%)	0.001
Atrial fibrillation	0 (0%)	2 (0.3%)	0.553
Stroke	7 (6.4%)	20 (3.2%)	0.103

^*^Data are presented as median (interquartile ranges) or number (percentage)

*CCVD* cardiocerebrovascular disease, *DM* diabetes mellitus, *HT* hypertension, *BAH* bilateral adrenal hyperplasia, *UHA* unilateral hyperaldosteronism, *SBP* systolic blood pressure, *DBP* diastolic blood pressure, *BMI* body mass index, *K* potassium, *Na* sodium, *PRA* plasma renin activity

## Discussion

In this study, we are the first to demonstrate the prevalence of newly diagnosed DM in PA. We found that 15.0% of PA patients had newly diagnosed diabetes, which was higher than the prevalence among those in the general population [3]. Previous studies have reported that the prevalence of diabetes ranges from 8.2 to 23% in the PA population [5, 20–22], in accordance with our findings. There are few studies on newly diagnosed diabetes in PA patients; however, we found that with elevated levels of aldosterone, the prevalence rates of newly diagnosed DM and abnormal glucose metabolism significantly increased. We found that elevated aldosterone increased the risk of DM and focused on newly diagnosed DM. It is vital that newly diagnosed DM patients with PA are assessed for cardiovascular damage compared to nondiabetic PA patients. To our knowledge, this is the first study of CCVD in PA patients with newly diagnosed DM. We found that the occurrence of CCVD in newly diagnosed diabetic patients was significantly higher than that in nondiabetic PA patients.

In our study, we found that the aldosterone level significantly influenced the glucose level. This result was in accordance with those of previous studies [10, 23–25]. The link we found between aldosterone and K^+^ may partially result from decreased insulin. In our study, we evaluated glucose and cortisol co-secretion (Supplementary Table S3), and we did not find a relationship, as reported previously [14, 26]. We found FBG and PBG slightly elevated in the group with unsuppressed 1 mg DST but not significantly. The cortisol and ACTH were similar between two groups however aldosterone was also slightly elevated in the group with unsuppressed 1 mg DST. Therefore, we deduced that aldosterone influenced glucose metabolism directly, and even though we adjusted for BMI, sex, duration of hypertension and blood pressure, the aldosterone level was an independent risk factor for DM. We found that aldosterone decreased insulin secretion during the OGTT; therefore, glucose increased, especially at the 120 min and 180 min time points. Hyperglycemic and euglycemic hyperinsulinemic clamp techniques for PA revealed that impaired glucose metabolism was due to a reduction in insulin secretion accompanied by increased insulin clearance [27]. This result was observed in PA patients without DM; however, the effect of aldosterone on insulin secretion and glucose is obvious in newly diagnosed PA patients with DM. Extra attention should be paid to newly diagnosed DM in PA patients.

Newly diagnosed DM means that patients have a shorter duration of high blood glucose and a higher chance of maintaining euglycemia and normotension with treatment. In general, high blood glucose levels were detected in 21% of all patients who died from ischemic heart disease and 13% of patients who died from stroke worldwide, with 84% of these deaths from CCVD [28]. In PA patients, excess aldosterone causes arterial hypertension and an increase in cardiovascular events, particularly atrial fibrillation [29]. One study found that DM was an independent risk factor for cardiovascular events and renal complications in PA patients [5]. Especially in PA, DM itself could increase cadio-cerebrovascular risk and renal complications in PA [5]. In our study, we found that this kind of damage occurred during the new-onset DM stage, reminding us to screen for DM in the diagnosis workflow of PA in the future. We also found renin activity elevated in patients with DM, meanwhile activation of the renin-angiotensin-aldosterone system may play a significant role in the development of insulin resistance and diabetes [30]. PA is a remediable hypertension, and patients with PA are at increased risk for CVD, cerebrovascular disease and arrhythmia compared with those with essential hypertension [31]. At the time of diagnosis, we found that CCVD in PA patients was related to DM and HT duration. This also emphasizes the importance of screening for DM at the first detection of PA; moreover, we need to screen for PA at the first detection of HT. PA screening in newly diagnosed hypertensive patients leads to good clinical outcomes [32]. In addition to screening for PA in newly diagnosed HT patients, we also need to screen for DM in newly diagnosed PA patients to prevent CCVD in PA patients.

There are some limitations of this study. This was a retrospective study, and a longer follow-up period may be needed to evaluate the relationship of glucose metabolism with CCVD in PA patients. In conclusion, the prevalence of newly diagnosed DM in PA patients was 15.0%, which was higher than that in the general population. Aldosterone was an independent risk factor for DM. PA patients with newly diagnosed DM had more CCV events, and CCVD positivity in PA patients correlated with DM and the duration of HT. Therefore, it is crucial to screen for DM in PA patients from the beginning.

## Supplementary information

Supplementary information
