# Supplementary material for: Newly diagnosed diabetes mellitus is a risk factor for cardiocerebrovascular events in primary aldosteronism
Source: Endocrine. 2022 Jul 29;77(3):519–26. doi: 10.1007/s12020-022-03095-8 (PMC9385765; doi:10.1007/s12020-022-03095-8)
Supplement: Supplementary file 1 — Supplementary information [file 12020_2022_3095_MOESM1_ESM.docx]

Supplementary

Table S1. Clinical characteristics of the study population according to serum aldosterone tertiles

|  | Tertile I  (-30.37) | Tertile II  (30.37-51.12) | Tertile III  (50.12-) | P for trend |
| --- | --- | --- | --- | --- |
| Number | 243 | 243 | 243 |  |
| Age(Years) | 47.0±10.9 | 44.8±10.5 | 47.0±10.8 | 0.987 |
| Gender(Male/Female) | 126/117 | 128/115 | 118/125 | 0.469 |
| Duration of HT(years) | 5(2,10) | 6(2,10) | 7(3,12) | 0.018 |
| SBP(mmHg) | 172±24 | 169±24 | 174±23 | 0.187 |
| DBP(mmHg) | 106±15 | 105±15 | 106±15 | 0.641 |
| BMI(kg/m²) | 24.8±3.7 | 24.6±3.5 | 24.0±3.3 | 0.012 |
| Serum cortisol (ug/dL) | 11.44±3.95 | 12.10±4.27 | 12.29±4.20 | 0.026 |
| ACTH(pg/mL) | 31.11±15.32 | 31.82±16.26 | 33.09±17.21 | 0.206 |
| Serum K^+^(mmol/L) | 3.15±0.44 | 3.10±0.42 | 2.86±0.46 | <0.001 |
| Serum Na^+^(mmol/L) | 141.64±2.86 | 141.68±2.31 | 142.44±2.87 | 0.001 |
| PRA(ng/ml/h) | 0.25(0.09,0.52) | 0.28(0.08,0.66) | 0.22(0.08,0.56) | 0.503 |
| ALD(pg/mL) | 22.83(18.61,26.25) | 40.03(34.57,45.98) | 71.97(60.29,95.36) | <0.001 |
| ARR(ng dl/ng ml h) | 75.09(41.79,257.33) | 153.93(62.32,500.41) | 347.78(140.01,1199.23) | <0.001 |
| FBG (mmol/L) | 5.13±0.58 | 5.26±0.70 | 5.27±0.72 | 0.023 |
| 2h PBG (mmol/L) | 7.36±2.38 | 8.05±2.92 | 8.37±3.11 | <0.001 |
| Insulin 0min  (μIU/ml) | 6.36(4.15,8.89) | 6.18(4.10,10.58) | 5.80(3.91,8.10) | 0.133 |
| Insulin 120min  (μIU/ml) | 44.42(28.83,77.18) | 50.48(29.54,85.52) | 47.08(29.81,73.17) | 0.557 |
| HOMA-IR | 1.42(0.93,2.04) | 1.40(0.91,2.48) | 1.33(0.89,1.83) | 0.521 |
| HbA1c | 5.31±0.51 | 5.41±0.59 | 5.44±0.90 | 0.107 |
| TG(mmol/L) | 1.42±0.98 | 1.44±0.85 | 1.42±1.01 | 0.950 |
| TC(mmol/L) | 4.26±0.83 | 4.26±0.82 | 4.35±0.72 | 0.187 |
| HDL(mmol/L) | 1.22±0.33 | 1.18±0.33 | 1.23±0.32 | 0.719 |
| LDL(mmol/L) | 2.54±0.68 | 2.59±0.72 | 2.62±0.60 | 0.182 |

*Continuous variables which obeyed normal distribution were shown as mean and standard deviation. Otherwise, median and inter-quartile range were considered.; categorical variables are expressed as frequencies and percentages.

Table S2 Clinical and Biochemical Parameters in CCVD-Positive and CCVD-Negative PA Patients

|  | CCVD-Positive PA Patients (N=44) | CCVD-Negative PA Patients (N=685) | P value |
| --- | --- | --- | --- |
| Age(years) | 53(44,59) | 47(38,54) | 0.001 |
| Gender(Male/Female) | 30/14 | 342/343 | 0.019 |
| Duration of HT (years) | 10(5,19) | 6(2,10) | <0.001 |
| Family history of HT | 33(75%) | 397(58%) | 0.026 |
| SBP(mmHg) | 175(159,197) | 170(159,180) | 0.210 |
| DBP(mmHg) | 105(100,120) | 101(100,115) | 0.381 |
| BMI(kg/m²) | 25.39(24.48,27.39) | 24.22(21.88,26.71) | 0.182 |
| Serum K^+^(mmol/L) | 3.01(2.82,3.41) | 3.02(2.80,3.32) | 0.540 |
| Serum Na^+^(mmol/L) | 142(140,145) | 142(140,144) | 0.614 |
| PRA(ng/ml/h) | 0.28(0.17,0.48) | 0.24(0.08,0.59) | 0.666 |
| Aldosterone(ng/dL) | 37.45(24.58,54.19) | 40.32(26.27,61.27) | 0.834 |
| BAH/UHA | 16/28 | 195/490 | 0.263 |
| DM,N% | 13(29.5%) | 96(14.0%) | 0.005 |
| Hypertriglyceridemia,N,% | 14(31.8%) | 173(25.3%) | 0.334 |
| Hypercholesteremia,N,% | 2(4.5%) | 27(3.9%) | 0.843 |

* Continuous variables are expressed as medians (interquartile ranges 25-75); categorical variables are expressed as frequencies and percentages

**Abbreviation: CCVD cardiocerebrovascular disease; HT, hypertension; SBP, systolic blood pressure; DBP, diastolic blood pressure; BMI, body mass index, K, potassium; Na, sodium; PRA, plasma renin activity; BAH, bilateral adrenal hyperplasia; UHA, unilateral hyperaldosteronism; DM, diabetes mellitus.

Table S3. The clinical characteristics and glucose metabolism of cohort patients stratified by 1mg DST

|  | 1mg<1.8  (N=344) | 1mg>1.8  (N=38) | P |
| --- | --- | --- | --- |
| Age(years) | 46(36,54) | 53(44,58) | 0.001 |
| Gender(Male/Female) | 192/152 | 13/25 | 0.011 |
| Duration of HT (years) | 6(2,10) | 10(5,15) | 0.025 |
| SBP(mmHg) | 168(153,180) | 165(150,180) | 0.399 |
| DBP(mmHg) | 102(97,115) | 100(97,110) | 0.394 |
| BMI(kg/m²) | 24.45(21.72,26.93) | 23.71(21.99,25.54) | 0.323 |
| Serum cortisol (ug/dL) | 11.26(8.45,14.23) | 11.00(9.16,15.21) | 0.953 |
| ACTH(pg/mL) | 28.15(20.87,38.96) | 23.63(18.74,34.69) | 0.072 |
| Serum K^+^(mmol/L) | 2.99(2.81,3.27) | 2.93(2.68,3.14) | 0.276 |
| Serum Na^+^(mmol/L) | 143(141,144) | 143(141,145) | 0.361 |
| PRA(ng/ml/h) | 0.25(0.08,0.63) | 0.16(0.06,0.48) | 0.201 |
| Aldosterone(ng/dL) | 40.93(26.10,64.75) | 48.19(33.02,61.89) | 0.237 |
| ARR(ng dl/ng ml h) | 191.96(66.30,539.05) | 231.26(79.41,1118.83) | 0.227 |
| FBG (mmol/L) | 5.16(4.83,5.53) | 5.28(4.91,5.84) | 0.202 |
| PBG(mmol/L) | 7.37(6.03,9.17) | 7.71(6.67,10.82) | 0.173 |
| Insulin 0min (μIU/ml) | 6.39(4.26,9.50) | 5.83(4.17,10.39) | 0.593 |
| Insulin 120min (μIU/ml) | 48.81(31.58,82.14) | 43.64(31.45,74.55) | 0.626 |
| HOMA-IR | 1.52(0.99,2.20) | 1.39(0.87,2.45) | 0.726 |
| HbA1c | 5.3(4.9,5.5) | 5.2(5.1,5.6) | 0.491 |
| DM% | 53(15.4%) | 7(18.4%) | 0.628 |
| Abnormal glucose metabolism% | 165(48.0%) | 22(57.9%) | 0.245 |
| TG (mmol/L) | 1.11(0.82,1.69) | 1.20(0.88,1.89) | 0.189 |
| TC (mmol/L) | 4.22(3.68,4.74) | 4.42(3.89,5.09) | 0.093 |
| HDL(mmol/L) | 1.13(0.95,1.40) | 1.16(1.02,1.29) | 0.689 |
| LDL (mmol/L) | 2.49(2.08,2.99) | 2.66(2.27,3.12) | 0.059 |
